# Relationship between FFR, CFR and coronary microvascular resistance – Practical implications for FFR-guided percutaneous coronary intervention

**DOI:** 10.1371/journal.pone.0208612

**Published:** 2019-01-07

**Authors:** Damien Garcia, Brahim Harbaoui, Tim P. van de Hoef, Martijn Meuwissen, Sukhjinder S. Nijjer, Mauro Echavarria-Pinto, Justin E. Davies, Jan J. Piek, Pierre Lantelme

**Affiliations:** 1 CREATIS, INSERM U1206, Université Lyon 1, INSA Lyon, Villeurbanne, France; 2 Department of Cardiology, Hôpital de la Croix-Rousse, Hospices Civils de Lyon, France; 3 AMC Heart Center, Academic Medical Center, University of Amsterdam, Amsterdam, The Netherlands; 4 Department of Cardiology, Tergooi Hospital, Blaricum, The Netherlands; 5 Department of Cardiology, Amphia Hospital, Breda, The Netherlands; 6 Imperial College London, London, United Kingdom; Universita degli Studi Magna Graecia di Catanzaro, ITALY

## Abstract

**Objective:**

The aim was threefold: 1) expound the independent physiological parameters that drive FFR, 2) elucidate contradictory conclusions between fractional flow reserve (FFR) and coronary flow reserve (CFR), and 3) highlight the need of both FFR and CFR in clinical decision making. Simple explicit theoretical models were supported by coronary data analyzed retrospectively.

**Methodology:**

FFR was expressed as a function of pressure loss coefficient, aortic pressure and hyperemic coronary microvascular resistance. The FFR-CFR relationship was also demonstrated mathematically and was shown to be exclusively dependent upon the coronary microvascular resistances. The equations were validated in a first series of 199 lesions whose pressures and distal velocities were monitored. A second dataset of 75 lesions with pre- and post-PCI measures of FFR and CFR was also analyzed to investigate the clinical impact of our hemodynamic reasoning.

**Results:**

Hyperemic coronary microvascular resistance and pressure loss coefficient had comparable impacts (45% and 49%) on FFR. There was a good concordance (*y* = 0.96 *x* − 0.02, *r*^2^ = 0.97) between measured CFR and CFR predicted by FFR and coronary resistances. In patients with CFR < 2 and CFR/FFR ≥ 2, post-PCI CFR was significantly >2 (p < 0.001), whereas it was not (p = 0.94) in patients with CFR < 2 and CFR/FFR < 2.

**Conclusion:**

The FFR behavior and FFR-CFR relationship are predictable from basic hemodynamics. Conflicting conclusions between FFR and CFR are explained from coronary vascular resistances. As confirmed by our results, FFR and CFR are complementary; they could jointly contribute to better PCI guidance through the CFR-to-FFR ratio in patients with coronary artery disease.

## Introduction

**Fig 1:**
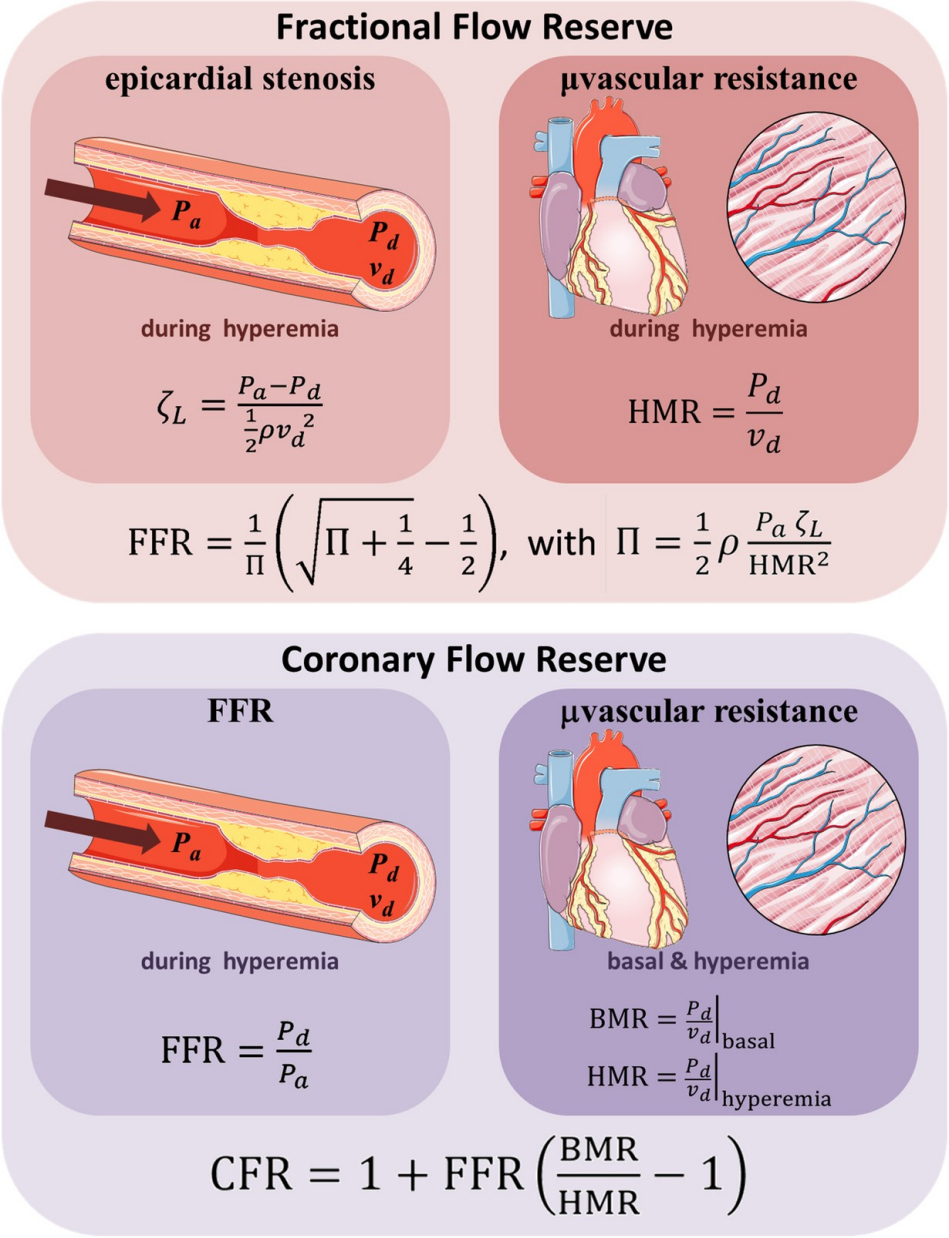
Theoretical expressions of FFR and CFR. FFR can be expressed as a function of a dimensionless parameter Π, which relates aortic pressure (*P*_*a*_), pressure loss coefficient (*ζ*_*L*_) and hyperemic microvascular resistance (HMR). CFR and FFR are interrelated through the basal-to-hyperemic microvascular resistance ratio.

Fractional flow reserve (FFR) is an invasive measure of the physiological significance of an epicardial coronary stenosis. Since coronary angiography is often insufficient in guiding percutaneous coronary intervention (PCI), FFR has gained wide acceptance for estimating whether a coronary lesion may cause myocardial ischemia [1]. FFR is defined as the ratio of distal (*P*_*d*_) to proximal (= aortic, *P*_*a*_) pressure (FFR = *P*_*d*_/*P*_*a*_) determined during pharmacologically-induced hyperemia (Fig 1). A lesion with an FFR ≤0.80 is generally judged ischemia-prone, whereas it is accepted that a lesion with an FFR >0.80 is unlikely to produce myocardial ischemia [2]. The impact of an epicardial stenosis on myocardial perfusion can alternatively be assessed by the Doppler- or thermodilution-derived coronary flow reserve (CFR). CFR denotes the myocardial reserve vasodilator capacity, defined as the ratio of maximal hyperemic coronary blood flow (CBF) to resting CBF [3]. CFR less than 2 is used to distinguish coronary lesions that are likely to trigger myocardial ischemia [4]. Although both FFR and CFR are aimed at identifying ischemia-prone lesions, they are discordant in ~30% of intermediate stenoses [5,6]. This disagreement does not reflect inaccuracy of either tool; but it illustrates that both FFR and CFR are not self-contained diagnostic criteria for a clear-cut decision of whether PCI is required. The FFR does not merely reflect the morphology of the stenosis but rather reveals its functional impact within its physiological surround [1]. Indeed, FFR depends not only upon the severity of the local lesion but also on hyperemic vascular resistance [7,8], and in a lesser but significant extent, on aortic pressure [9,10]. Although the relationship between FFR and CFR has not been unequivocally established, it is known to be mostly modulated by the coronary microvascular resistance [8,11]. For this reason, it has been suggested that intracoronary pressures and flows should be measured simultaneously to distinguish the impact of the focal stenosis from that of downstream coronary resistance for a better therapeutic decision in patients with coronary lesions [8,12,13].

Our first objective was to explicitly determine once and for all the physiological parameters that drive FFR. The second objective was to expound how the coronary microvascular resistances intermingle FFR and CFR. The purpose was to make it clear 1) why a significant stenosis can have an FFR greater than 0.8, and 2) why so-called discordances between FFR and CFR have been reported in recent clinical studies. Based on our theoretical rationale, our third objective was to exemplify the need of both FFR and CFR to better guide percutaneous coronary intervention.

To know how FFR, CFR and coronary physiology are interrelated, we sought to describe the hemodynamic relationships that precisely link these fundamental parameters by using simple mathematical representations. This could help reconsidering the classical 0.8-FFR threshold in situations where an epicardial stenosis exists concomitantly with significant coronary diffuse disease and/or microvasculature disorder. More importantly, an explicit formulation will shed some light on how factors external to the locally stenotic region may quantitatively impact FFR and CFR, before and after PCI, allowing one to enhance interpretation of combined pressure-flow measurements and their application in clinical practice. From a clinical viewpoint, the hemodynamic models that we derived also highlight the need to use CFR in conjunction with FFR to predict the potential benefit of PCI for a given stenosis.

## Methods

### Hemodynamic background

#### Theoretical expression of FFR

The pressure loss coefficient (*ζ*_*L*_, also called Euler number) is a fluid-dynamics-based dimensionless parameter that describes the relationship between the pressure drop throughout a flow field and the corresponding kinetic energy. It mainly reflects pressure losses due to wall friction and/or turbulence. In particular, it can quantify the severity of a flow constriction and has been used in the context of epicardial coronary stenoses [14,15]:

$$\zeta_{L}=\frac{P_{a}-P_{d}}{\frac{1}{2}\rho v_{d}^{2}},$$ (1)

where *v*_*d*_ is the distal blood velocity and *ρ* stands for blood density. In the particular (uncommon) case of a non-elongated axisymmetric stenosis, *ζ*_*L*_ can be related to the stenosis severity (see S1 File) and is not flow-dependent. Flow dependence of *ζ*_*L*_, however, generally occurs in extended stenoses as a result of wall friction [16], which makes it unfeasible to estimate from geometry only. In practice, computational fluid dynamics is thus required to relate *ζ*_*L*_ to the stenosis geometry [17]. The hyperemic coronary vascular resistance in terms of flow velocity is defined by [8]

$$HMR={\frac{P_{d}}{v_{d}}|}_{hyperemia}.$$ (2)

HMR corresponds to the microvascular resistance downstream of the focal stenosis. It reflects the resistance due to downstream diffuse disease (if any) in series with the resistance of the coronary microvasculature (Fig 1). By using a dimensional analysis [18] and involving the three linearly independent physical quantities that drive FFR (*P*_*a*_, *ζ*_*L*_, and HMR), FFR can be related to this dimensionless parameter:

$$\Pi =\frac{1}{2}\rho \frac{P_{a}\zeta_{L}}{{HMR}^{2}}.$$ (3)

Developing Π (see S1 File) yields a simple quadratic equation (Π FFR^2^ + FFR − 1) = 0, whose solution gives a generalized expression of FFR (Fig 1):

$$FFR=\frac{1}{\Pi }\left(\sqrt{\Pi +\frac{1}{4}}-\frac{1}{2}\right).$$ (4)

In this study, we used this expression to investigate how, and to which extent, independent hemodynamic parameters can affect FFR. As a matter of fact, Eq 4 shows that FFR decreases when Π increases *i*.*e*. when: 1) *ζ*_*L*_ increases, 2) HMR decreases, and/or 3) *P*_*a*_ increases. It follows that FFR decreases in the following situations: 1) when the focal stenosis becomes more severe; 2) when diffuse coronary disease is less significant; 3) when aortic pressure increases. Eq 4 corroborates clinical observations [8,10]. In comparison with previously published models [7,9], it has the advantage to provide an explicit association between FFR and three linearly independent hemodynamic parameters (*i*.*e*. three degrees of freedom).

### Relative effects of P_a_, HMR and ζ_L_ on FFR

We showed through Eq (4) that FFR totally depends upon three independent physiological parameters. These parameters may have different impacts on FFR, depending on their absolute values and ranges. Using the differential of FFR, it can be shown that the amount of FFR variation (ΔFFR) around the 0.8-threshold value is related to *P*_*a*_, *ζ*_*L*_ and HMR variations (*i*.*e*. Δ*P*_*a*_, Δ*ζ*_*L*_ and ΔHMR) as follows (see S1 File):

$$\Delta FFR=\frac{2}{15}(2\frac{\Delta HMR}{HMR}-\frac{\Delta P_{a}}{P_{a}}-\frac{\Delta \zeta_{L}}{\zeta_{L}}).$$ (5)

This equation predicts how an incremental change in any of the three parameters *P*_*a*_, *ζ*_*L*_ and HMR affects FFR. As will be confirmed later in the Results, not only the severity of stenosis (*ζ*_*L*_) but also the coronary microvascular resistance (HMR) have a major impact on FFR.

#### Theoretical expression of CFR

The coronary flow reserve (CFR) represents the ratio of maximal hyperemic CBF over basal CBF [3]. At first it was thought that there was a one-to-one correspondence between FFR and CFR in coronary pathophysiology. This misconception led to supposedly discordant FFR-vs.-CFR conclusions when quantifying coronary physiology. It is now well accepted that the coronary microvascular resistance comes into play in the CFR-FFR relationship [7]. The latter, however, is still poorly identified. Assuming that aortic pressure is unchanged between baseline and hyperemia, as generally happens with adenosine, it can be shown (in the S1 File) that the coronary flow reserve can be approximated by the following expression:

$$CFR=1+FFR\left(\frac{BMR}{HMR}-1\right),$$ (6)

where BMR and HMR are the basal and hyperemic coronary microvascular resistances, respectively (Fig 1). Eq (6) allows one to offer an unambiguous explanation on previous clinical observations that have reported “discordances” between FFR and CFR. More importantly, it shows that FFR alone or CFR alone cannot be used unequivocally since their relationship is clearly influenced by the coronary microvascular resistances. Eq (6) can be rewritten as follows:

$$\frac{CFR}{FFR}=\frac{1}{FFR}+\frac{BMR}{HMR}-1.$$ (7)

Recent clinical studies have shown that elevated coronary microvascular resistances predict poor outcomes in FFR-guided PCI [19,20]. The above expression pointedly shows that the joint information combining FFR and coronary microvascular resistances (right side) is directly related to the CFR-to-FFR ratio (left side). In this retrospective study, we analyzed the clinical impact of the CFR-to-FFR ratio in predicting post-PCI CFR, as explained later.

### Invasive CFR and FFR measurements

To validate Eqs (4), (5) and (6), we retrospectively reevaluated 299 coronary stenoses in 228 anonymized patients reported in [8]. Note that we also considered the lesions with FFR<0.60, although they were rejected in [8]. The patients were referred for intracoronary assessment of at least one intermediate coronary lesion. The baseline characteristics of these patients are presented in the Table 1 of reference [8]. Exclusion criteria were listed in [8]. Distal and proximal coronary pressures, as well as distal blood velocities, were measured subsequently during basal and hyperemic conditions. Intracoronary pressures were monitored with a 0.014” guide wire (Volcano, San Diego, USA). Coronary flow velocities were determined using a FloWire Doppler guide wire (Volcano, San Diego, USA). Coronary hyperemia was induced by intracoronary administration of adenosine (20–40 μg). From the recorded pressure and velocity waveforms, FFR was calculated as the ratio of averaged distal to aortic pressure during maximum hyperemia; BMR (HMR) was determined by the ratio of averaged distal coronary pressure to averaged distal velocity during basal (hyperemic) conditions; *ζ*_*L*_ was estimated by the ratio between averaged trans-stenotic pressure difference and distal kinetic energy (see Eq 1) during maximum hyperemia; CFR was calculated as the ratio of hyperemic to basal averaged peak velocities. The procedures were approved by the medical ethical committee of the Academic Medical Center (Amsterdam, The Netherlands) and all patients gave written informed consent.

To validate Eq (6) and investigate the clinical value of combined CFR and FFR, a second dataset of 75 lesions in 67 anonymized patients was reanalyzed retrospectively. This dataset included coronary pressure and velocity measurements before and after PCI [21]. The latter were obtained simultaneously using a dual sensor-equipped guide wire (ComboWire, Volcano, San Diego, USA). Adenosine was administered by intravenous continuous infusion in 43 stenoses (140 μg/kg per minute) and by intracoronary bolus in 32 stenoses (60 μg). The same dose was used before and after intervention. Coronary intervention was performed at the operator’s discretion based on usual clinical care, including angiographic and noninvasive findings. Measurements were repeated after angioplasty at the same location as pre-angioplasty. At the end of each recording, the pressure sensor was returned to the catheter tip to avoid any pressure drift. The measurements were repeated when drift was identified. An adequate flow velocity envelope was obtained in all patients permitting the calculation of flow-based indices. The baseline characteristics of these patients are presented in the Table 1 of reference [21]. The procedures were approved by the ethical committees of the Academic Medical Centre (Amsterdam, the Netherlands) and Imperial College (London, UK) and all patients gave written informed consent.

### Data analysis

A first series of analyses was carried out using the first dataset (299 lesions). FFR variations around 0.8 (ΔFFR) were estimated from Eq (5) and compared with the measured FFR variations using a Bland-Altman analysis. This analysis was performed around the critical value 0.8, using the subpopulation whose FFR ranged between 0.7 and 0.9 (*N* = 168). The relative variations of *P*_*a*_, *ζ*_*L*_ and HMR (2^nd^ term of Eq 5) were calculated as Δ*x*/*x* = (*x* − median(*x*))/*x*, with *x* being *P*_*a*_, *ζ*_*L*_ or HMR. The actual ΔFFR was determined by (FFR − 0.8). To point up the respective impacts of coronary microvascular resistance and stenosis severity on FFR, FFR was also displayed in a scatter diagram as a function of HMR and log(*ζ*_*L*_), assuming a mean aortic pressure of 95 mmHg. CFR was finally estimated from FFR and basal (BMR) and hyperemic (HMR) coronary microvascular resistances (Eq 6). It was compared with the CFR measured by Doppler guide wire using a linear regression and a Bland-Altman analysis.

The impact of PCI on CFR was evaluated in the 75 lesions in which pre- and post-intervention pressure and flow measurements were available. Eq (7) reveals that the CFR/FFR ratio might have some prognostic value. The prognostic ability of CFR/FFR in predicting post-PCI CFR>2 was thus evaluated through a receiver operating characteristic (ROC) analysis. The optimal cut-off point was determined by maximizing the Cohen’s kappa statistic, which turned out to be almost equal to 2. The 75 lesions were separated in three groups: #1) CFR > 2; #2) CFR ≤ 2 and CFR/FFR ≥ 2; #3) CFR ≤ 2 and CFR/FFR < 2. The cut-off value of 2 was chosen to comply with the ROC analysis. For each group, the pre- and post-PCI CFR were compared using a one-tailed paired t-test to test the alternative hypothesis that CFR mean was greater after than before PCI. Post-PCI CFR were also analyzed using a one-tailed one-sample t-test to test the alternative hypothesis that post-PCI-CFR mean was greater than 2.

## Results

### Impact of HMR, *ζ*_*L*_ and P_a_ on FFR

**Fig 2:**
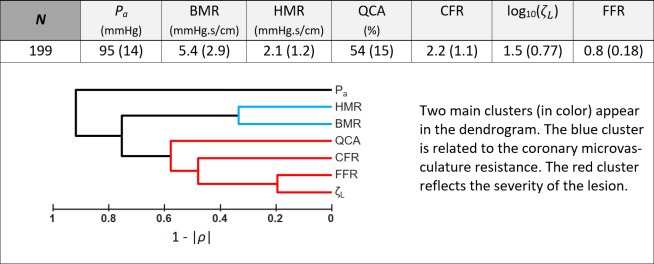
Hemodynamic parameters of the 1^st^ dataset (n = 199). *P*_*a*_. = aortic pressure; BMR = basal coronary microvascular resistance; HMR = hyperemic coronary microvascular resistance; QCA = diameter reduction (%) by quantitative coronary analysis; *ζ*_*L*_ = pressure loss coefficient; FFR = fractional flow reserve. The table reports median ± robust standard deviations. The dendrogram represents the average-link similarities returned by an agglomerative hierarchical clustering. Modulus of the Spearman’s rank correlation coefficient (*ρ*) was used as a distance metric; it reflects the proximity between two objects by measuring at what point they are similar. Reported values = median (interquartile range).

**Fig 3:**
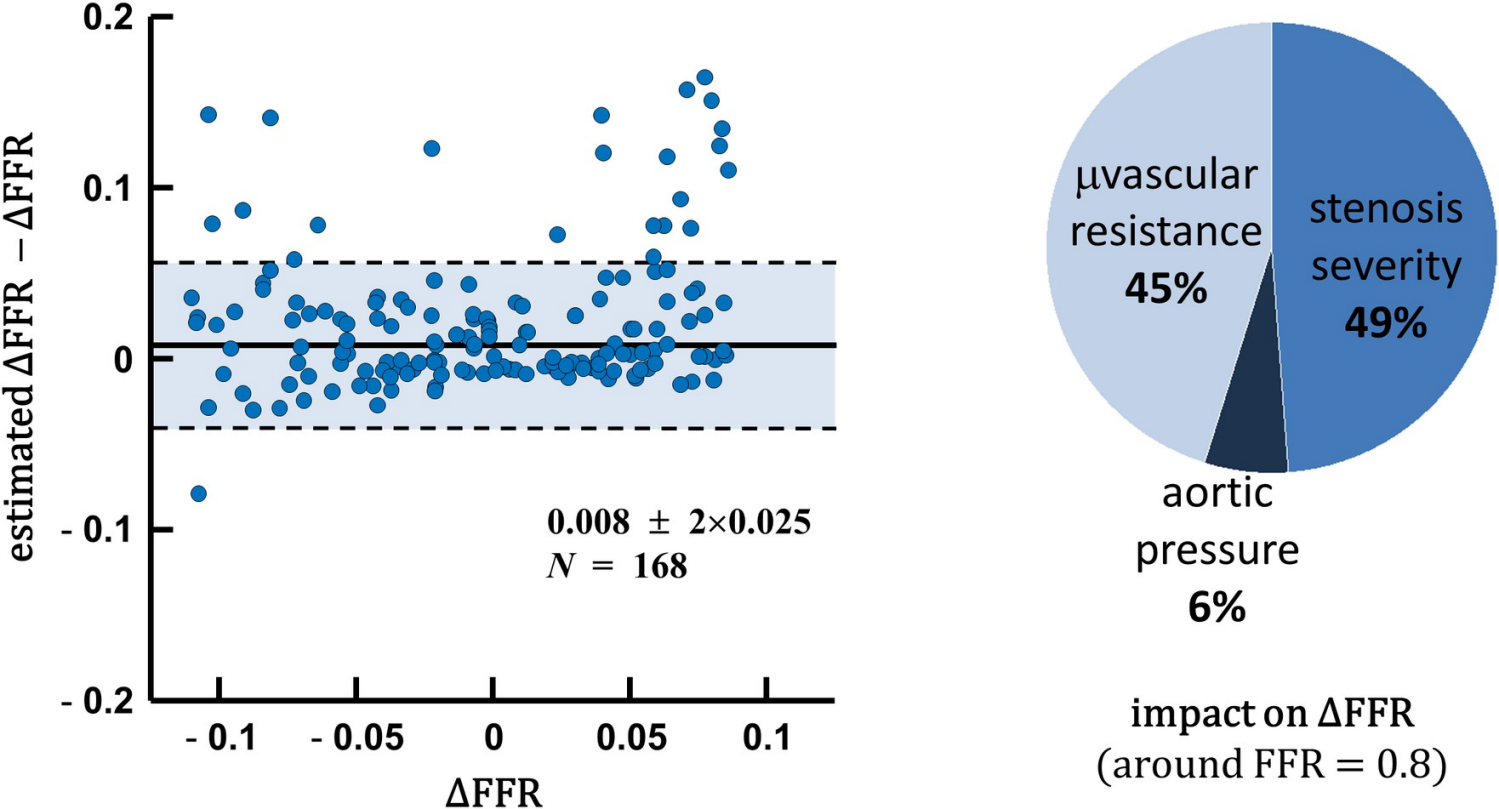
FFR variations around 0.8. Left panel: estimated *vs*. actual FFR variations around the 0.8-threshold value (Eq 5). Right panel: respective impacts of hyperemic microvascular resistance, stenosis severity and aortic pressure on FFR variation.

**Fig 4:**
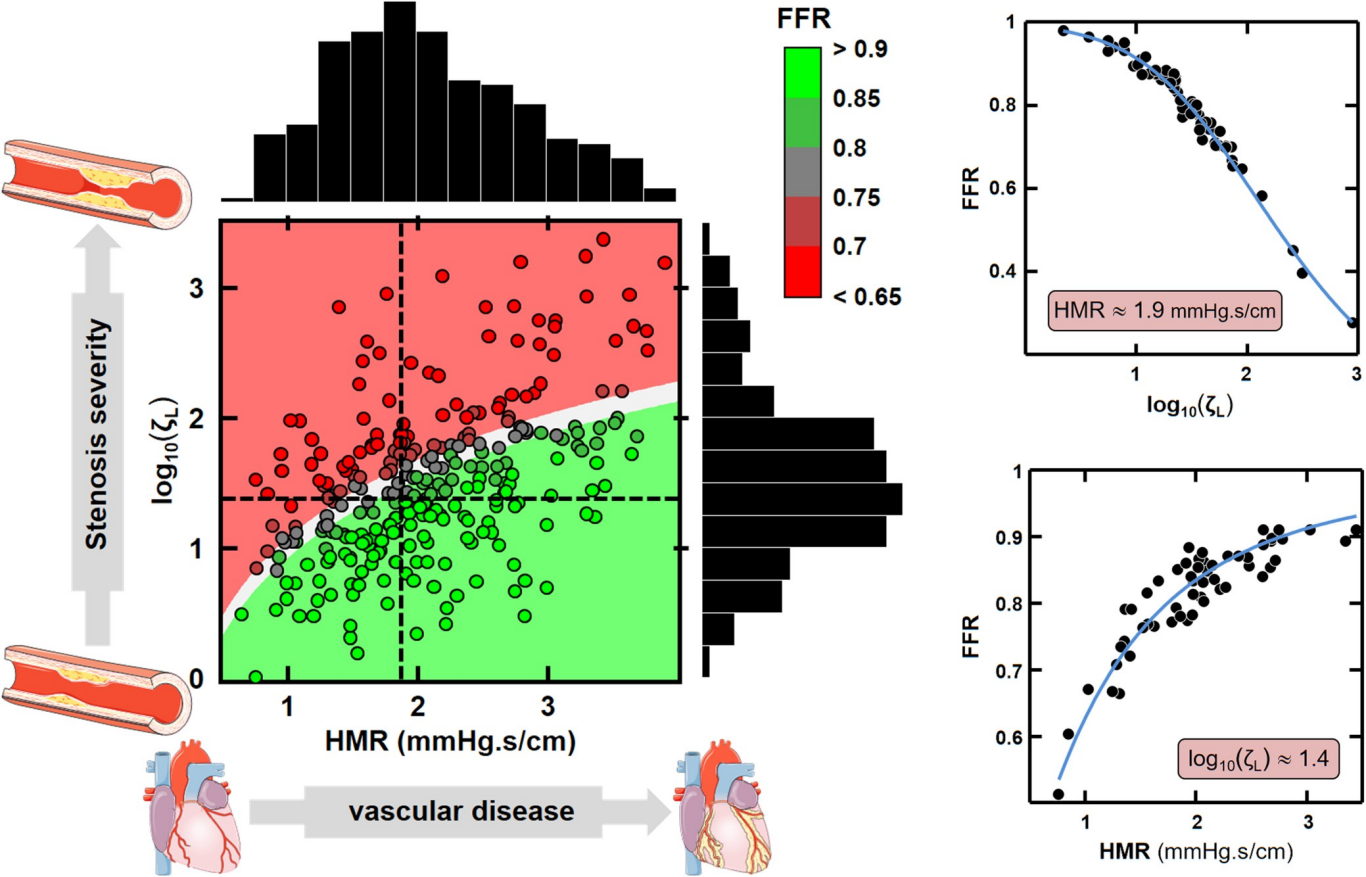
Relationship between HMR, ζ_L_ and FFR. ***Left panel***: The dot colors represent the FFR measured by the pressure guide wire. The colored background is the theoretical FFR (Eq 4) assuming a proximal pressure of 95 mmHg (red: FFR<0.75; grey: 0.75<FFR<0.8; green: FFR>0.8). The dashed lines identify the modes of *ζ*_*L*_ and HMR distributions. ***Right panel***: Independent effects of *ζ*_*L*_ and HMR on FFR around their respective modes. HMR and log_10_(*ζ*_*L*_) were fixed at 1.9 ± 0.19 mmHg/cm/s and 1.4 ± 0.14, respectively. The blue curves are theoretical (Eq 4).

There was a strong Spearman’s rank correlation (*ρ* = -0.8) between FFR and *ζ*_*L*_ (see dendrogram in Fig 2). Fig 3 shows that Eq 5 was a good predictor of FFR variation around the critical value 0.8. The median absolute error was 0.008, with a robust standard deviation of 0.025. The pie chart indicates that HMR and *ζ*_*L*_ had comparable impact (45% and 49%) on FFR variation in this subpopulation (whose FFR was around 0.8), whereas aortic pressure had lesser effect (6%). Fig 4 further reveals that FFR was highly dependent on both HMR and *ζ*_*L*_. These observations are in line with those obtained by Morris *et al*. by computational fluid dynamics [22].

### Relationship between FFR and CFR

**Fig 5:**
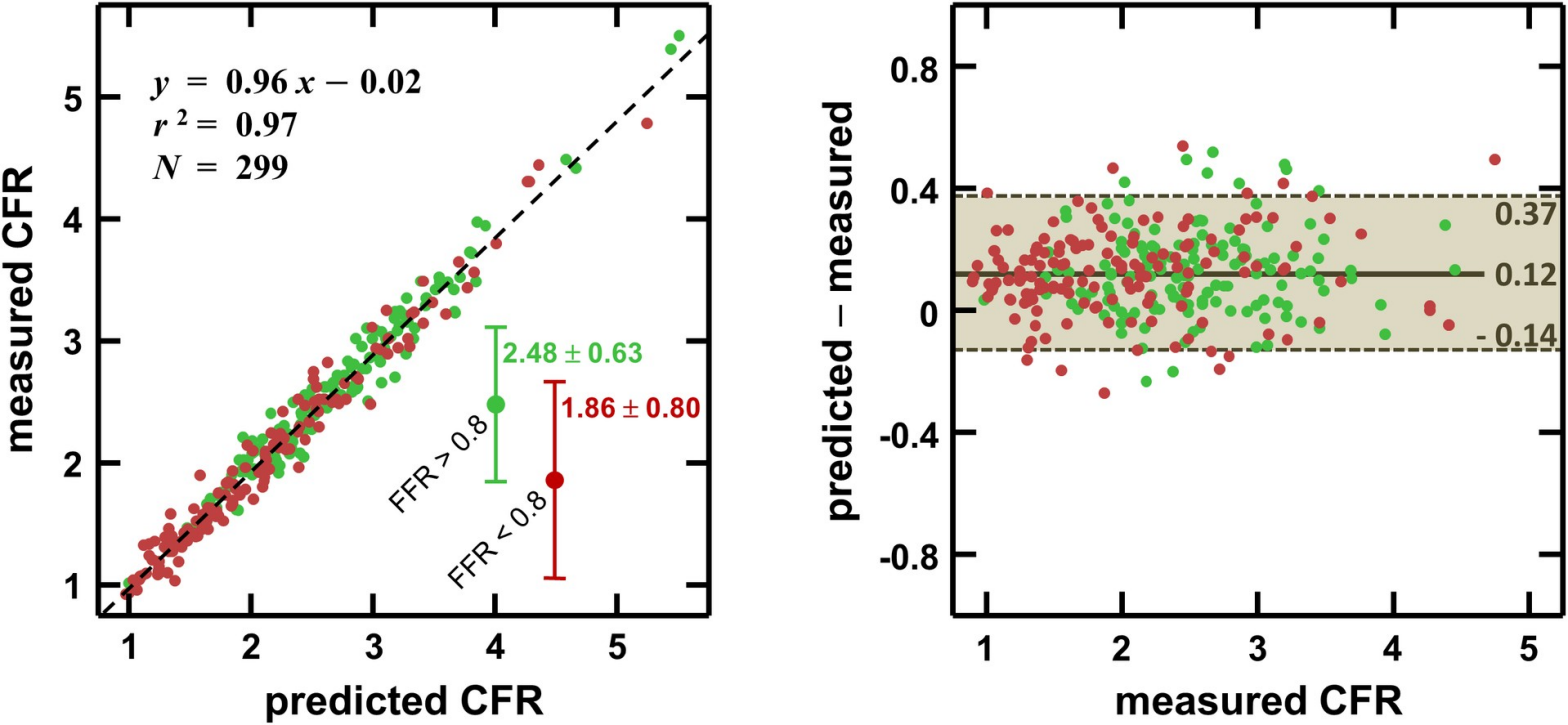
Predicted *vs*. measured CFR. CFR was predicted from Eq (6). The green (red) dots correspond to lesions with FFR greater (less) than 0.8. The inset represents the corresponding median CFR values ± robust standard deviation.

**Fig 6:**
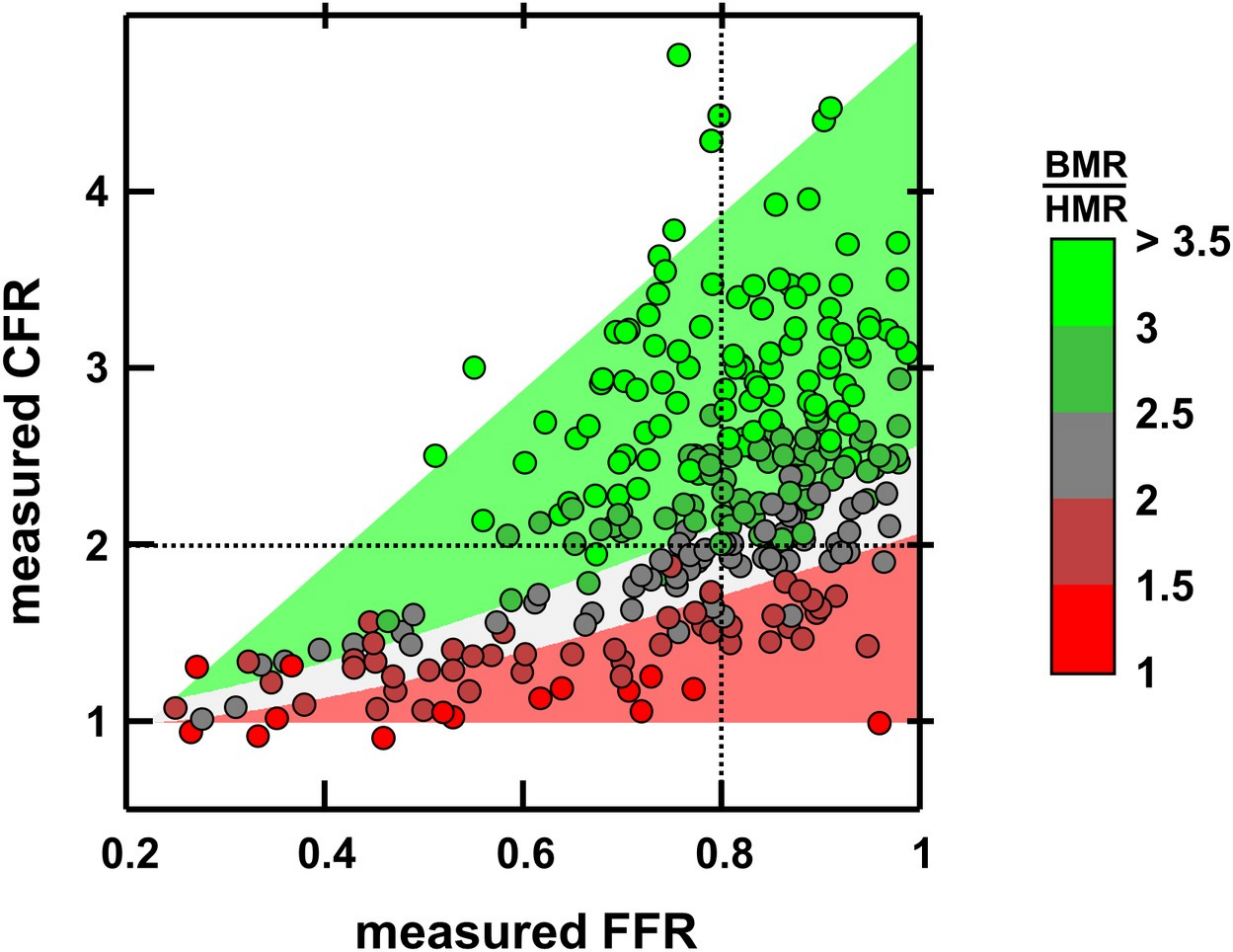
Relationship between FFR, CFR and (BMR/HMR). CFR and FFR are related through the ratio of basal to hyperemic vascular resistances (BMR / HMR); see Eq (6). The dot colors represent the measured BMR-over-HMR ratios. The colored background illustrates the theoretical ratio (red: ratio<2; grey: 2<ratio<2.5; green: ratio>2.5).

The linear regression between predicted (see Eq 6) and measured pre-PCI CFR returned *y* = 0.96 *x −* 0.02, *r*^2^ = 0.97 (Fig 5). The median absolute error was 0.12, with a robust standard deviation of 0.13, which shows that Eq 6 was a good predictor of CFR prior to PCI. The triangle-shaped CFR-FFR relationship is illustrated in Fig 6. This figure confirms that CFR and FFR are mostly related through the basal-to-hyperemic microvascular-resistance ratio.

### Pre-PCI CFR/FFR as a prognostic marker?

**Fig 7:**
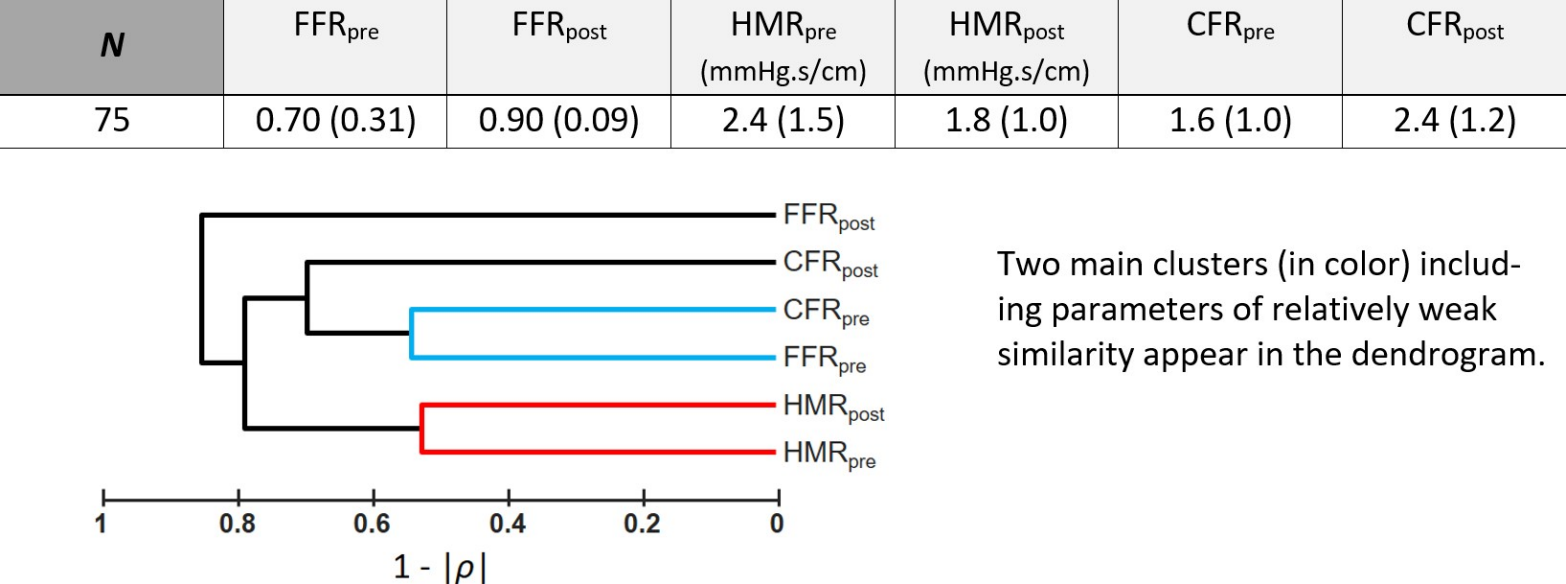
Hemodynamic parameters of the 2^st^ dataset (n = 75). Same acronyms as in Fig 2. Subscripts “pre” and “post” refer to pre- and post-revascularization, respectively. Reported values = median (interquartile range).

**Fig 8:**
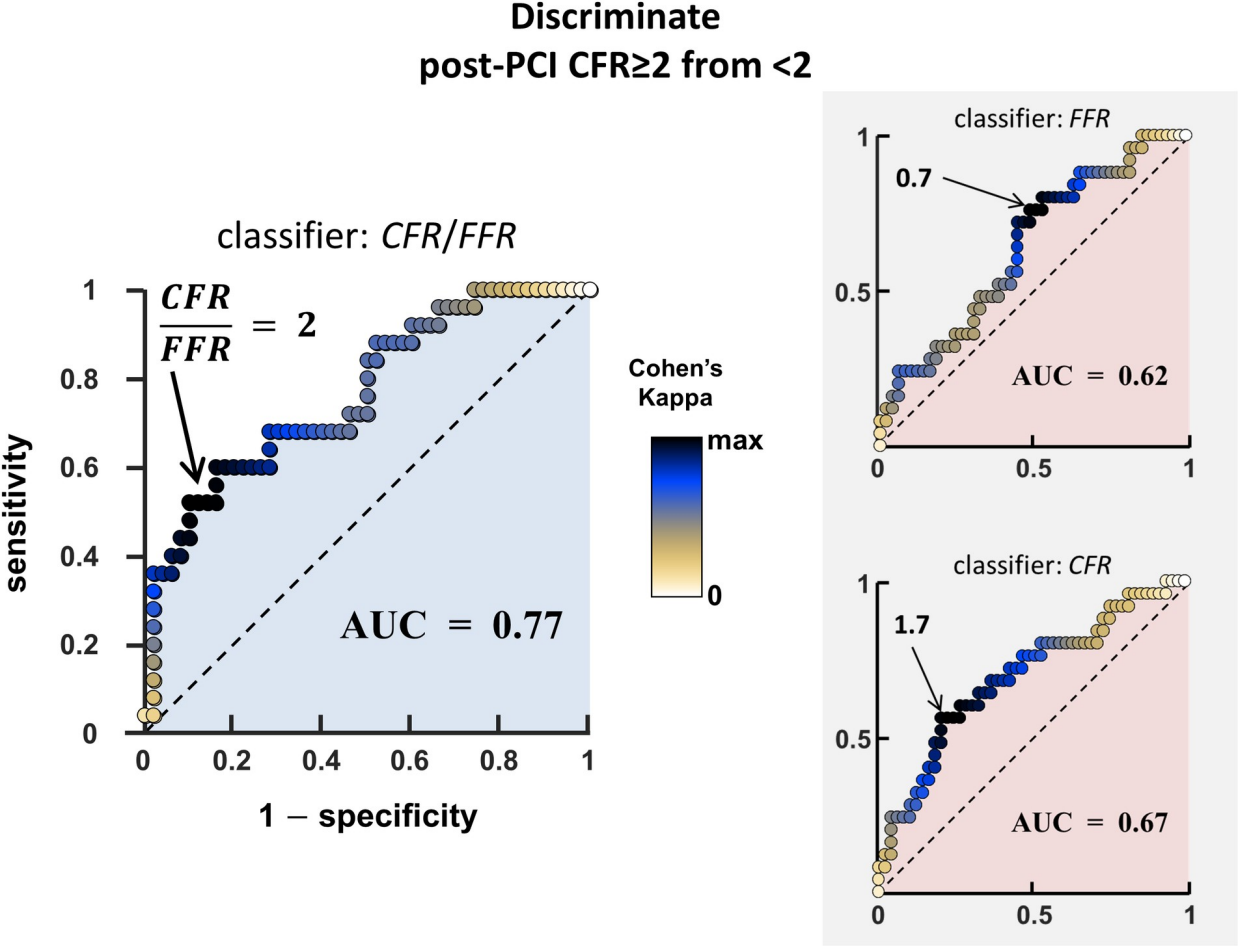
ROC analysis. Accuracy of the CFR-to-FFR ratio to discriminate post-PCI CFR>2 from post-PCI CFR<2. AUC = area under the ROC curve. The colored disks represent the Cohen’s kappa statistic. The ROC curves of the FFR and CFR classifiers are also represented for comparison.

**Fig 9:**
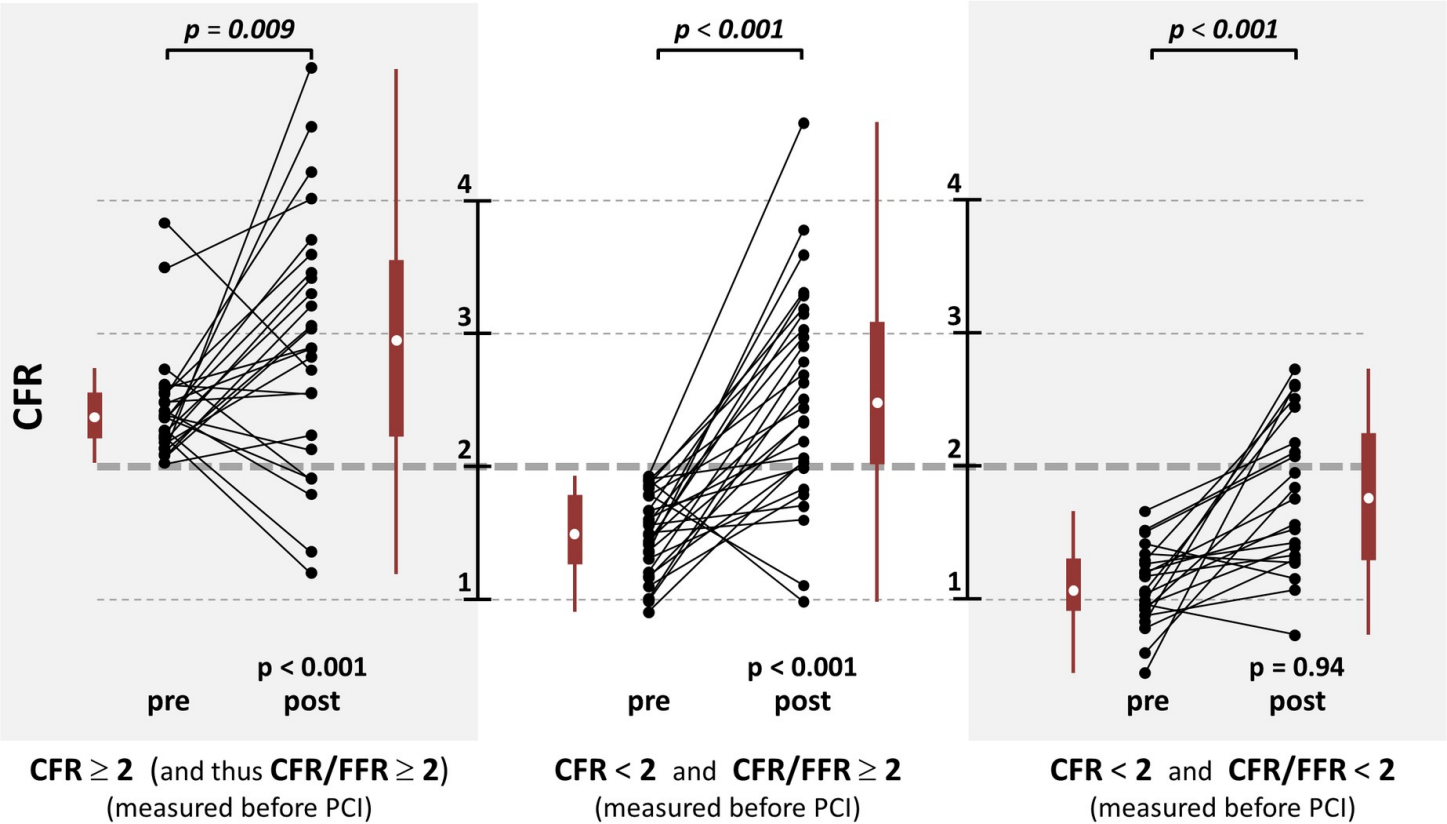
Measured CFR before *vs*. after PCI. The white dots in the boxplots represent the median values. Non-italicized p values (1^st^ rows) refer to the one-sample right-tailed t-tests with the alternative hypothesis that post-PCI-CFR mean was greater than 2. Italicized p values (top) refer to the one-sample right-tailed t-tests with the alternative hypothesis that post-PCI-CFR mean was greater than pre-PCI-CFR mean.

Weak to modest Spearman’s rank correlations (|*ρ*| < 0.5) were observed between pre- and post-PCI FFR, HMR and CFR (see dendrogram in Fig 7). The area under the ROC curve (AUC) was 0.77 (Fig 8), which denoted a fair-to-good discrimination of CFR/FFR in predicting a post-PCI CFR greater than 2. The optimal cut-off determined by the Cohen’s kappa statistic was 1.93 ≈ 2. This cut-off yielded a specificity and sensitivity of 87% and 56%, respectively. CFR increased significantly after PCI in the three groups (Fig 9). In groups #1 and #2 (CFR/FFR ≥ 2), post-PCI CFR was significantly greater than 2 (p < 0.001), whereas it was not (p = 0.94) in group #3 (CFR/FFR < 2).

## Discussion

A number of clinical studies have reported that FFR is governed not only by the severity of the epicardial stenosis but also by the downstream coronary vascular resistance. The latter has also been shown to be the main source of discordance between FFR and CFR in roughly 30% of patients with intermediate lesion. In this study, we provided mechanistic evidences to support previous clinical observations. We derived explicit equations to solve the resistance dilemma of FFR. We also demonstrated mathematically the interplay of FFR and CFR. These equations were validated retrospectively using pressure and velocity coronary data. The main findings are that 1) FFR can be completely expressed by three linearly independent hemodynamic variables, and 2) FFR and CFR are directly related through the basal-to-hyperemic microvascular resistance ratio. More importantly, this study also calls attention to conflicting conclusions that may be noticed between CFR and FFR. According to the CFR *vs*. FFR relationship, clinical conflicts between FFR and CFR can actually be predicted from basic hemodynamics. Strictly speaking, there is no discordance as such since FFR and CFR are distinct in nature. These fundamental parameters are rather complementary and it is anticipated that they could jointly contribute to better PCI guidance. We conclude that focal coronary stenoses should be assessed by taking their surrounding environment into account, and their functional assessment should preferentially be based on both CFR and FFR. It is expected that the CFR-to-FFR ratio could be of prognostic relevance in predicting post-PCI CFR and thus has the potential to optimize patient treatment.

### Epicardial stenosis and downstream microvascular resistance contribute equally to FFR

The expression relating FFR to the dimensionless Π parameter demonstrates that FFR is governed by the 1) stenotic pressure loss coefficient, 2) downstream hyperemic microvascular resistance and 3) aortic pressure. The involvement of these variables has been already reported [8,10,15]; their impact on FFR, however, was not clearly established since no explicit model was available. Although aortic pressure might influence FFR in particular pathophysiological conditions, such as hypotension [10], its effect was small in our study (Fig 3). Fig 4 further confirmed that FFR was mostly regulated by *ζ*_*L*_ and HMR, both controlling the coronary pressure-flow relationship. The pressure loss coefficient *ζ*_*L*_ depends on the stenotic flow constriction and length [16]: constricted sections induce flow separation, an unstable process that causes irreversible pressure loss; elongated stenoses also make wall friction significant, an additional source of pressure loss. Other factors such as wall curvature and tortuosity can also increase pressure losses [23]. This multifactorial association explains the moderate correlation observed between the diameter-based severity and *ζ*_*L*_ (Spearman’s rank correlation coefficient |ρ|<0.5; see dendrogram in Fig 2. See also S1 Fig in the S1 File). Hyperemic microvascular resistance HMR involves the coronary microvasculature downstream from the lesion. It increases in situations such as diffuse coronary disease, acute myocardial infarction [24], cardiac hypertrophy [25] or reduced vasodilatory capacity [26]. Our mathematical model establishes that FFR increases with increasing HMR, all other parameters being equal, as reported by Meuwissen *et al*. [12]. It follows that an epicardial stenosis may appear “less severe” (increased FFR) when significant downstream microvascular dysfunction is present. In this study, HMR and *ζ*_*L*_ contributed equally to FFR modulation around the critical 0.8-threshold. As emphasized in previous clinical investigations, our findings confirm mathematically and experimentally that microvascular resistance, and thus flow or velocity measurements, must be considered when interpreting FFR. As discussed below, this could be achieved by considering CFR in the diagnostic algorithm.

### FFR and CFR are interrelated through coronary microvascular resistance

As depicted by Eq 6, the microvascular resistance dictates the relationship between CFR and FFR. We found that CFR can be approximated by {1 + FFR (BMR/HMR-1)}, where BMR and HMR are the basal and hyperemic coronary microvascular resistances. This expression shows that CFR decreases and/or FFR increases as HMR decreases. This is concordant with Meuwissen *et al*. [11] who reported that HMR was lower (1.9 *vs*. 2.4 mmHg/cm/s) in patients with CFR ≥ 2 and FFR < 0.75 (*i*.*e*. CFR/FFR > 2.67) than those with CFR < 2 and FFR ≥ 0.75 (*i*.*e*. CFR/FFR < 2.67). Coronary microvascular resistance is thus in large part responsible for FFR-CFR diagnostic conflicts. Normal-CFR abnormal-FFR situations may occur with high BMR-to-HMR ratios (>2.5) exclusively (Fig 6, left upper quadrant, 22.1% of the patients), whereas abnormal-CFR normal-FFR states are seen only if BMR-to-HMR ratio is low (< 2.5, right lower quadrant in Fig 6, 8.7%). This explains why a so-called discordance between FFR and CFR occurs in roughly 30% of the patients with intermediate stenoses [5,6]. FFR and CFR are often clinically discordant simply because they are not in one-to-one correspondence. An abnormal CFR cannot reliably discriminate significant epicardial stenosis from non-obstructive vascular dysfunction [27]. In like manner, FFR cannot reckon the diffuse disease that may exist concomitantly with a focal stenosis. Since FFR and CFR are both interwove with microvascular resistance, they cannot intend to be alike, but should rather be considered as complementary diagnostic parameters.

### Functional assessment based on CFR and FFR

**Fig 10:**
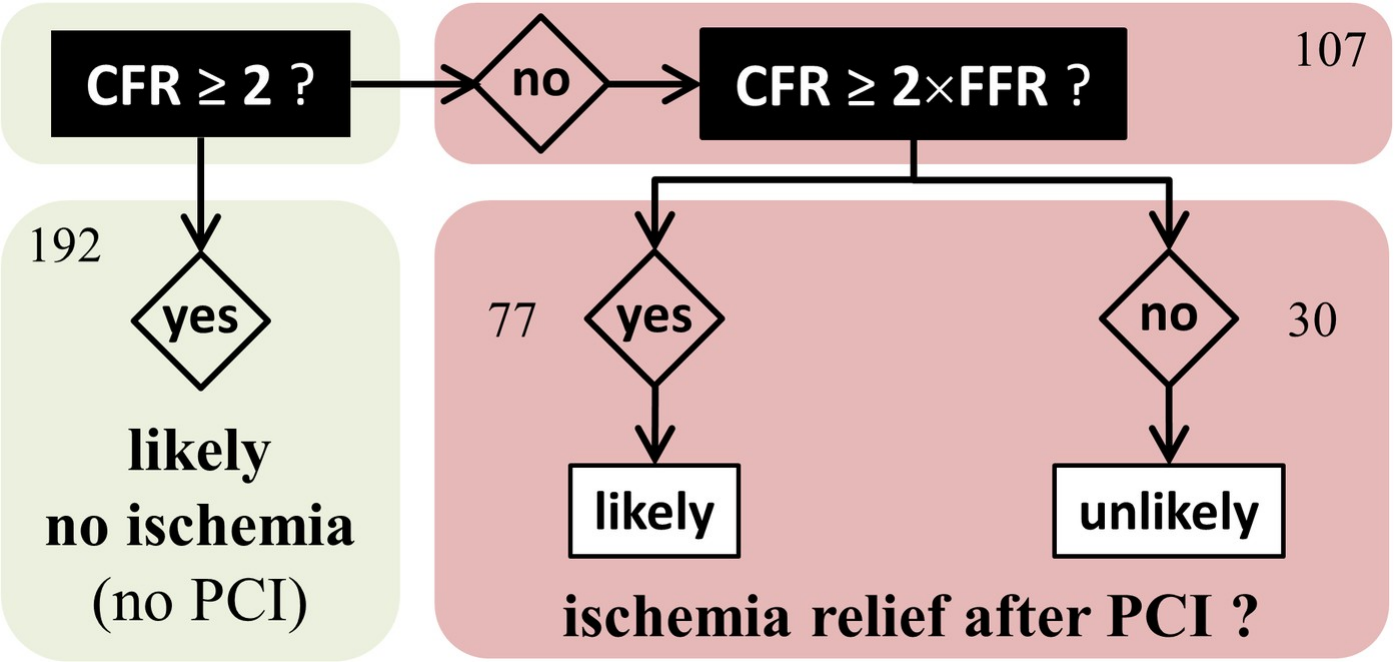
Likelihood of ischemia relief after PCI. This flowchart speculates on ischemia relief by revascularization based on our theoretical and experimental findings. The numbers represent the occurrences among the 299 lesions.

As discussed above, our findings document that there is an interplay of FFR, CFR and microvascular resistance. From our biomechanical reasoning, it is expected that FFR alone should not promote PCI in a number of situations. When assessing the function of a coronary stenosis, the physician must answer two questions: 1) is the coronary lesion likely to induce ischemia?, 2) if so, will a PCI reduce the risk of myocardial ischemia? The well-accepted 0.8 cut-off value for FFR theoretically ensures that a significant increase in CFR can be expected after PCI. If an abnormal FFR is documented with an intermediate lesion, FFR-guided PCI can thus be beneficial and a gain in CFR is to be targeted. This precautionary measure, however, does not ensure ischemia relief if CFR/FFR<2 since post-PCI CFR could remain smaller than 2, as reflected by group #3 in Fig 9 (3^rd^ column). This situation may occur for example in diabetic patients [28]. In daily clinical practice, ischemia may not be evaluated before catheterization. In such condition, FFR alone cannot independently foresee the hemodynamic effect of PCI. When coronary flow reserve is relatively preserved, FFR may be misleading as blood flow remains sufficient to meet myocardial demand. It has been concordantly shown that a preserved CFR excludes high-risk CAD with a high negative predictive value [27]. In this circumstance, symptoms are unlikely to be improved after revascularization; revascularization could thus be safely deferred in patients with CFR >2 (group #1 in Fig 9, 1^st^ column). With regard to patients with abnormal CFR (i.e. <2), pre-PCI CFR/FFR should ideally be >2 to substantially relieve ischemia and increase the benefit of PCI (Fig 9, 2^nd^ vs. 3^rd^ column). Significant focal stenosis and vascular dysfunction coexist in patients with CFR/FFR < 2 (group #3 in Fig 9, 3^rd^ column). In such patients, PCI can possibly be of little advantage in terms of ischemia relief, and therapeutic tactics targeting the diffuse disease might be an option. To conclude, a focal stenosis should be assessed by taking its surrounding environment into account, and its functional assessment should preferentially be based on both CFR and FFR (Fig 10). This strategy should be investigated in a prospective outcome study. Interestingly, an ongoing prospective multicenter trial (DEFINE-FLOW, NCT02328820) is evaluating the prognostic value of combined pressure and flow measurements in coronary stenosis. DEFINE-FLOW aims at investigating whether revascularization in low-FFR high-CFR lesions can be deferred. The hypothesis is that lesions with an intact CFR (≥ 2.0) can be reasonably treated with medical therapy despite a reduced FFR (≤ 0.8). Lesions with preserved CFR and reduced FFR will thus receive optimal medical therapy. Only lesions with a reduction in both CFR and FFR will be treated with PCI. In the same vein, and according to our findings (see Figs 8 and 10), we believe that the CFR-to-FFR ratio could be of diagnostic relevance.

### Limitations

Although FFR has gained worldwide recognition, it turns out that a critical parameter is still missing to potentially predict post-PCI outcome. In this study, we mathematically confirmed that the missing link is CFR, or alternatively the basal-to-hyperemic vascular resistance ratio. Unfortunately, CFR is rarely measured in clinical practice. To complicate matters, CFR is limited by its dependence upon heart rate [29] and requires reliable Doppler or temperature measures. It is presently challenging and time-consuming to measure coronary flow by Doppler or thermodilution approaches. Efforts thus must be made to design robust technologies to allow clinicians to get reproducible and simultaneous pressure- and flow-based parameters in hopes of better guiding PCI.

The proposed model and the experimental data were based on single stenoses in series with downstream microvascular resistance. Extrapolation of our conclusions to serial stenoses should be treated with caution. We also examined intermediate lesions, as evaluated by coronary angiography. Clinical situations with critical stenoses, endothelial dysfunction or microvascular disease were not investigated. These particular conditions could be considered as extrema within the hemodynamic range observed clinically. Note also that our results were interpreted around FFR = 0.8 and CFR = 2 since we chose the generally accepted cut-off values. Whether other threshold values should be assigned to optimize PCI diagnosis must be investigated prospectively in a large cohort.

### Clinical relevance

Despite the clinical importance of FFR and CFR in the assessment of the coronary physiology, their interrelationship and their relationships with other hemodynamic parameters have remained poorly understood. In the present study, we have posed analytical equations to explicate supposedly-conflicting observations that were reported in previous clinical studies. We have explicitly confirmed that the evaluation of epicardial stenosis severity by FFR is in large part disguised by the coronary microvascular resistance. The FFR-CFR relationship that we derived also clearly demonstrates that the pretended discordance between CFR and FFR is governed by the coronary microvascular resistance. These hemodynamic expressions reveal that not only pressure-based but also flow-based measurements should be considered in interventional cardiology practice. In particular, we anticipate that the CFR-to-FFR ratio could be of major clinical relevance in optimizing individual patient treatment.

## Conclusion

We demonstrated that both stenosis severity and coronary microvascular resistance modulate FFR and CFR. This study contributes to the growing awareness of the significance of coronary resistance and supports the observations that considering CFR can aid clinical decision-making during coronary angiography. From mathematical and clinical observations, it is anticipated that the CFR-to-FFR ratio may have a substantial prognostic ability in predicting post-PCI ischemia relief. Development of simple tools to measure FFR and CFR simultaneously should be promoted.

## Supporting information

S1 File Mathematical derivations of the hemodynamic formulas. (DOCX)

S2 File Matlab (version R2017b) figures. Use the Matlab function “get” to retrieve the original data. Read the Matlab documentation (mathworks.com/help/matlab/ref/get.html) for details.
(ZIP)

## Abbreviations

BMR: basal coronary microvascular resistance
CBF: coronary blood flow
CFR: coronary flow reserve
FFR: fractional flow reserve
HMR: hyperemic coronary microvascular resistance
*P_a_*: aortic pressure
PCI: percutaneous coronary intervention
*P_d_*: distal pressure
*ρ*: blood density
*v_d_*: distal blood velocity
*ζ_L_*: pressure loss coefficient
