# Supplementary material for: Relationship between FFR, CFR and coronary microvascular resistance – Practical implications for FFR-guided percutaneous coronary intervention
Source: PLoS One. 2019 Jan 7;14(1):e0208612. doi: 10.1371/journal.pone.0208612 (PMC6322913; doi:10.1371/journal.pone.0208612)
Supplement: S1 File — (DOCX) [file pone.0208612.s001.docx]

**Relationship between FFR, CFR and coronary microvascular resistance –**

**Practical implications for FFR-guided percutaneous coronary intervention**

Damien Garcia *et al.*

**– Supporting Information S1 –**

**• *Derivation of FFR***

We derived an expression for FFR from the dimensionless Π parameter (Equation 3, main manuscript). For this purpose, we used a basic dimensional analysis. It must be noticed that a number of different tuples of linearly independent variables could be used to derive FFR. Our objective, however, was to explain why FFR is dependent on 1) **the pressure loss coefficient**, 2) **aortic pressure**, and 3) **microvascular resistance**. It was thus natural to use the triplet (𝜁𝐿, Pa, HMR). Of note, these three hemodynamic parameters are linearly independent, which is required when using the Π-theorem.

| The dimensionless $\Pi$ parameter (see Eq. 3) can be developed as follows:  $\Pi=\frac{1}{2}\rho\frac{P_{a}\zeta_{L}}{\mathrm{HMR}^{2}}=\frac{1}{2}\rho\frac{P_{a} \left[ \left( P_{a}-P_{d} \right)/\left( \frac{1}{2}\rho v_{d}^{2} \right) \right]}{\left[ P_{d}/v_{d} \right]^{2}}=\frac{P_{a}\left( P_{a}-P_{d} \right)}{{P_{d}}^{2}}=\frac{{P_{a}}^{2}}{{P_{d}}^{2}}-\frac{P_{a}}{P_{d}}=\frac{1}{\mathrm{FFR}^{2}}-\frac{1}{\mathrm{FFR}}$. | (A1) |
| --- | --- |

(A1) can be rewritten using the following quadratic equation

| $\Pi\mathrm{FFR}^{2}+FFR-1=0$. | (A2) |
| --- | --- |

Solving for FFR leads to

| $\mathrm{FFR}=\frac{1}{\Pi}\left( \sqrt{\Pi+\frac{1}{4}}-\frac{1}{2} \right)$. | (A3) |
| --- | --- |

This equation can be developed using a Taylor series about FFR = 0.8 (*i.e.* $\Pi=$5/16):

| $\mathrm{FFR}=0.8-\frac{32}{75}\left( \Pi-\frac{5}{16} \right)+\frac{1}{2} \frac{1073}{1286}\left( \Pi-\frac{5}{16} \right)^{2}$. | (A4) |
| --- | --- |

The absolute difference between expressions (A3) and (A4) is less than 0.01 when FFR is in the range [0.7 – 0.9].

**• *FFR variation around FFR = 0.8***

| FFR depends on three independent variables. The differential of FFR leads to:  $\Delta FFR\approx\frac{\partial\mathrm{FFR}}{\partial\mathrm{HMR}}\Delta HMR+\frac{\partial FFR}{\partial P_{a}}\Delta P_{a}+\frac{\partial FFR}{\partial\zeta_{L}}\Delta\zeta_{L}$, | (A5) |
| --- | --- |

which can be rewritten as

| $\Delta FFR\approx\frac{\partial\mathrm{FFR}}{\partial\Pi}\left( \frac{\partial\Pi}{\partial HMR}\Delta HMR+\frac{\partial\Pi}{\partial P_{a}}\Delta P_{a}+\frac{\partial\Pi}{\partial\zeta_{L}}\Delta\zeta_{L} \right)$. | (A6) |
| --- | --- |

From the expression of $\Pi$ (Eq. 3), it follows that

| $\Delta FFR\approx\Pi\frac{\partial\mathrm{FFR}}{\partial\Pi}\left( -2\frac{\Delta HMR}{\mathrm{HMR}}+\frac{\Delta P_{a}}{P_{a}}+\frac{\Delta\zeta_{L}}{\zeta_{L}} \right)$. | (A7) |
| --- | --- |

Since, from (A4), $\Pi=\frac{5}{16}$ and $\frac{\partial\mathrm{FFR}}{\partial\Pi}=-\frac{32}{75}$ around FFR = 0.8, (A7) becomes

| $\Delta FFR\approx\frac{2}{15}\left( 2\frac{\Delta HMR}{\mathrm{HMR}}-\frac{\Delta P_{a}}{P_{a}}-\frac{\Delta\zeta_{L}}{\zeta_{L}} \right)$. | (A8) |
| --- | --- |

**• *Relationship between CFR and FFR***

CFR represents the ratio of maximal hyperemic CBF over resting (basal) CBF [1]. Expressing CFR in terms of velocities and multiplying by resting ($P_{d}{/P}_{a}$) yields:

| $\mathrm{CFR}\left( \frac{{P_{d}}_{\{basal\}}}{{P_{a}}_{\{basal\}}} \right)=\frac{{v_{d}}_{\{hyper\}}}{{v_{d}}_{\{basal\}}}\frac{{P_{d}}_{\{basal\}}}{{P_{a}}_{\{basal\}}}=\left( \frac{{P_{d}}_{\{hyper\}}}{{P_{a}}_{\{basal\}}} \right)\frac{{P_{d}}_{\{basal\}}/{v_{d}}_{\{basal\}}}{{P_{d}}_{\{hyper\}}/{v_{d}}_{\{hyper\}}}=\left( \frac{{P_{d}}_{\{hyper\}}}{{P_{a}}_{\{basal\}}} \right)\frac{\mathrm{BMR}}{\mathrm{HMR}}$ | (A9) |
| --- | --- |

Assuming that aortic pressure is unchanged between baseline and hyperemia (i.e. ${P_{a}}_{\{hyper\}}={P_{a}}_{\{basal\}}$, as generally happens with adenosine), (A9) becomes

| $\mathrm{CFR}\left( \frac{{P_{d}}_{\{basal\}}}{{P_{a}}_{\{basal\}}} \right)=\mathrm{FFR}\frac{\mathrm{BMR}}{\mathrm{HMR}}$. | (A10) |
| --- | --- |

To simplify equation (A10), we want to withdraw the resting ($P_{d}{/P}_{a}$) term by assuming that it depends on both CFR and FFR exclusively. To this end, we consider the behavior of Equation (A10) as $\mathrm{FFR}$ approaches 0 or 1.

When $\mathrm{FFR}$ approaches 1, resting ($P_{d}{/P}_{a}$) also approaches 1. As a result, (A10) shows that $\mathrm{CFR}\to\mathrm{FFR}\left( \mathrm{BMR}/\mathrm{HMR} \right)$ when $FFR\to1$.

On the other hand, $\mathrm{CFR}$ tends to 1 (i.e. no coronary reserve) if $\mathrm{FFR}$ approaches 0 (near-occluding stenosis). As a result, $\mathrm{CFR}\to1$ when $FFR\to0$.

A simple $\mathrm{CFR}$ expression that meets these two boundary conditions is the following

| $\mathrm{CFR}=1+\mathrm{FFR}\left( \frac{\mathrm{BMR}}{\mathrm{HMR}}-1 \right)$. | (A11) |
| --- | --- |

**• *Relationship between*** $\boldsymbol{\zeta}_{\boldsymbol{L}}$ ***and stenosis severity for a non-elongated axisymmetric stenosis***

The averaged pressure loss induced by an arterial stenosis is mainly due to the downstream flow divergence and wall friction [2]. For a non-elongated stenosis, losses induced by wall friction are negligible with respect to those produced by divergence. In that case, the pressure loss coefficient is similar to that of an aortic stenosis [3]:

| $\zeta_{L}= \left( \frac{D^{2}}{d^{2}}-1 \right)^{2}$, | (A12) |
| --- | --- |

where $D$ and $d$ are the non-stenotic and stenotic diameters. Since the severity of a stenosis (in percent) is $p=100\left( 1-d/D \right)$, the pressure loss coefficient of a non-elongated axisymmetric stenosis can be written as:

| $\zeta_{L}= \frac{p\left( 200-p \right)^{2}}{\left( 100-p \right)^{4}}$. | (A13) |
| --- | --- |

**• *Relationship between stenosis severity and*** $\boldsymbol{\zeta}_{\boldsymbol{L}}$

The following Figure 1A depicts the link (Eq. A13) between stenosis severity (in %) and the decimal logarithm of pressure loss coefficient. Note that this relationship is true only for a non-elongated stenosis (orifice plate model). The dots represent the data measured in patients (QCA and pressure wire). The correlation coefficient between stenosis severity and log_10_$\left( \zeta_{L} \right)$ was *r* = 0.55, which confirms that the hemodynamic property of a focal stenosis cannot be accurately predicted from QCA-derived inner diameters.

| 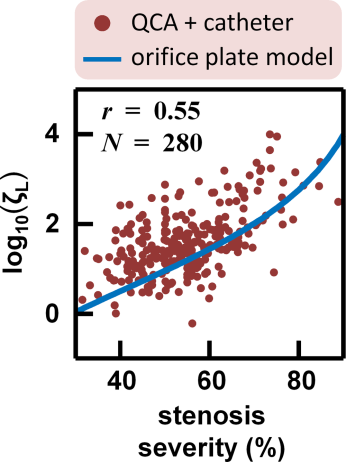 | | | |
| --- | --- | --- | --- |
|  | **Figure 1A – *Relationship between stenosis severity and*** $\boldsymbol{\zeta}_{\boldsymbol{L}}$. Orifice plate model and actual data. |  |  |

**• *Relationship between FFR and CFR***

The data presented in Figure 3 in the main text can also be displayed by the following Figure 2A. It illustrates the interplay of FFR, BMR/HMR and CFR.

| 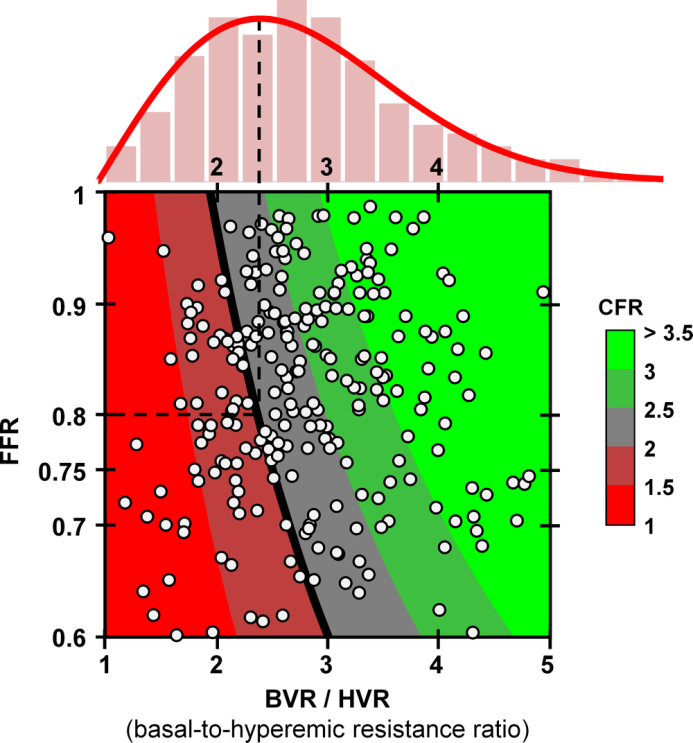 | | | |
| --- | --- | --- | --- |
|  | **Figure 2A – *Relationship between FFR, CFR and (BMR*/*HMR)***. CFR and FFR are related through the ratio of basal to hyperemic vascular resistances (BMR / HMR); see equations (6) and (A11). The colored background illustrates the theoretical relationship between CFR, FFR and (BMR/HMR). The thick solid line corresponds to CFR=2. The vertical dashed line identifies the mode of the BMR-over-HMR distribution; note that it crosses the line FFR = 0.8 at CFR = 2. |  |  |

**• *References***

[1] De Bruyne B, Sarma J. Fractional flow reserve: a review. Heart 2008;94:949–959. doi:10.1136/hrt.2007.122838.

[2] Young DF, Cholvin NR, Roth AC. Pressure drop across artificially induced stenoses in the femoral arteries of dogs. Circ Res 1975;36:735–743. doi:10.1161/01.RES.36.6.735.

[3] Garcia D, Pibarot P, Dumesnil JG, Sakr F, Durand L-G. Assessment of aortic valve stenosis severity. A new index based on the energy loss concept. Circulation 2000;101:765–771. doi:10.1161/01.CIR.101.7.765.
